# GLRB‐Related Hyperekplexia Presenting as Neonatal Seizure‐Like Events

**DOI:** 10.1111/jpc.70484

**Published:** 2026-06-29

**Authors:** Bruno Antunes Contrucci, Hugo Matos Bandeira, Sofia Tonin Angonese, Marcela Lopes de Almeida, Rafaela Pichini de Oliveira, Vitor Tumas, Ana Paula Andrade Hamad, Guillermo Andrey Ariza Traslaviña, Carla Andrea Cardoso Tanuri Caldas

**Affiliations:** ^1^ Department of Neurology Ribeirão Preto Medical School, University of São Paulo Ribeirão Preto Brazil

Hyperekplexia is a rare neurogenetic disorder caused by impaired glycinergic neurotransmission, classically characterized by exaggerated startle responses and generalized stiffness [1, 2, 3]. In the neonatal period, paroxysmal startle events may closely mimic epileptic seizures, frequently creating diagnostic uncertainty and potentially delaying appropriate treatment [4, 5, 6]. Although sustained stiffness is traditionally considered a defining feature of hyperekplexia, increasing clinical and genetic characterization has expanded the recognized phenotypic spectrum of the disorder [2, 3, 4].

Here, we report a genetically confirmed case of GLRB‐related hyperekplexia presenting with exaggerated startle responses and neonatal seizure‐like events in the absence of sustained generalized stiffness.

## 1 Case Presentation

We present a male infant with an unremarkable perinatal history and no evidence of hypoxia or neonatal complications. During the neonatal period, he developed a brief paroxysmal episode characterized by transient hypertonia and limb rigidity, initially raising concern for a neonatal seizure. Although electroencephalography showed no ictal correlate and demonstrated only nonspecific background abnormalities, a loading dose of phenobarbital was administered because of the clinical concern for seizure activity.

Neurological examination revealed global hypotonia with reduced primitive reflexes. In contrast, the glabellar reflex was markedly exaggerated and non‐habituating, accompanied by head retraction in response to tactile stimuli. No persistent generalized stiffness was observed during wakefulness or handling beyond the initial isolated neonatal event.

Brain MRI was unremarkable, and metabolic investigations were normal. Maternal and neonatal intoxication were excluded. Given the combination of global hypotonia and exaggerated startle responses, a genetic aetiology was suspected. Whole genome sequencing identified a homozygous pathogenic variant in *GLRB* (NM_000824.5:c.634C>T; p.Arg212Ter), consistent with hyperekplexia type 2 (OMIM #614619). Parental segregation analysis demonstrated heterozygosity in both parents, supporting autosomal recessive inheritance.

During follow‐up, the patient exhibited global developmental delay. At 4 months of age, he later developed clinically and electrographically confirmed focal epileptic seizures. Episodes were characterized by tonic posturing involving the right upper limb and cephalic version associated with upward ocular deviation, recurring on three occasions. Additional episodes consisted of isolated ocular deviation and left cephalic version. EEG demonstrated background disorganization with multifocal epileptiform paroxysms, without evidence of a focal structural aetiology on neuroimaging. These events were considered electroclinically distinct from the exaggerated startle responses and transient neonatal rigidity previously observed. Treatment with phenobarbital (5 mg/kg/day) was initiated, resulting in adequate seizure control. Because exaggerated startle responses persisted, clonazepam (0.05 mg/kg/day) was subsequently introduced, with partial clinical improvement.

**FIGURE 1:**
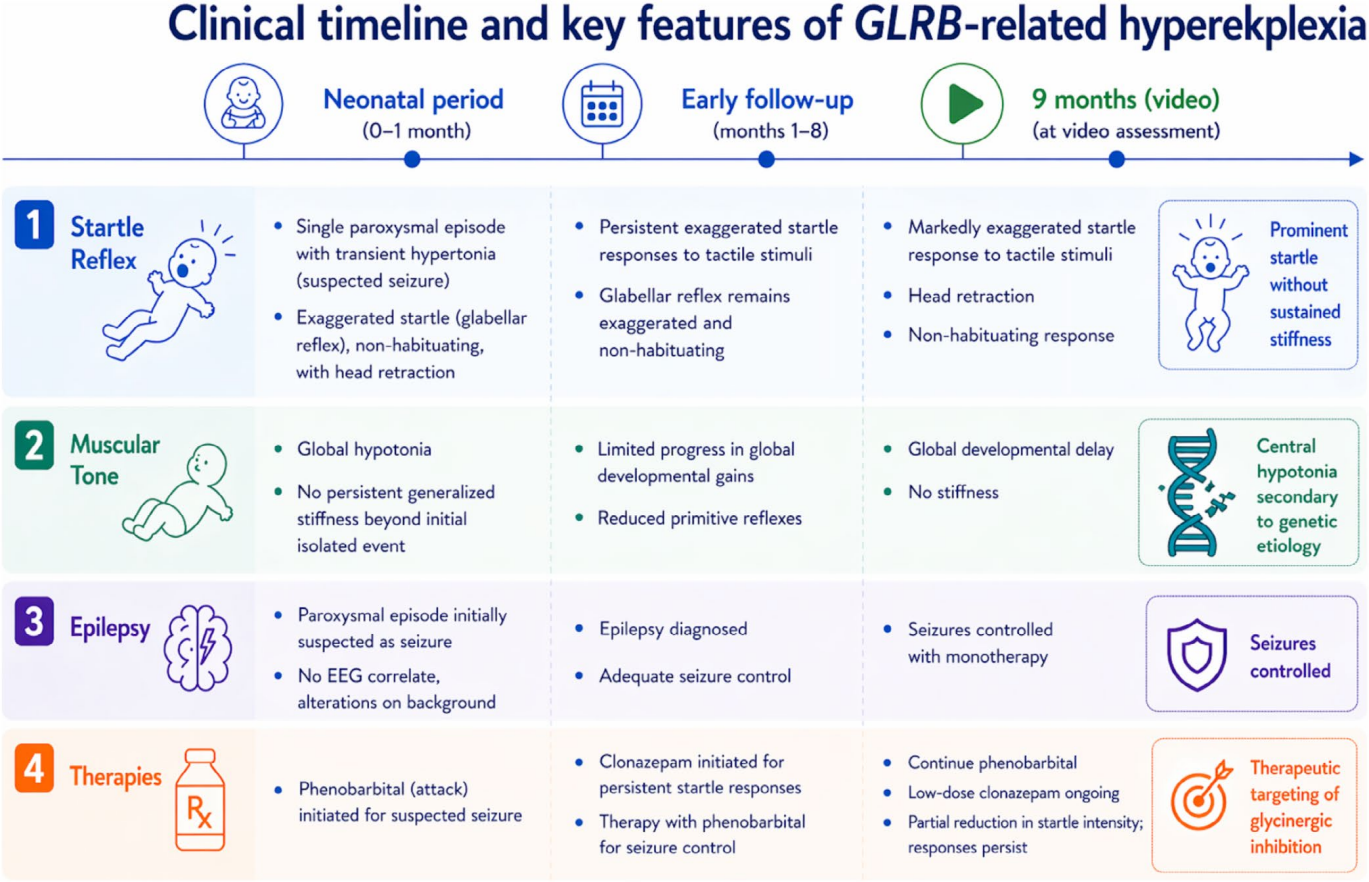
Chronological clinical evolution summarizing neonatal seizure‐like presentation, exaggerated startle responses, diagnostic workup and therapeutic interventions in GLRB‐related hyperekplexia.

At 9 months of age, persistent global hypotonia with incomplete head control was observed, requiring alternative feeding. Video examination at this stage revealed a markedly exaggerated startle response consistently triggered by tactile stimuli and associated with head retraction (Video S1). The response was non‐habituating and occurred in the absence of sustained generalized stiffness. Motor development remained delayed, with limited spontaneous movements and impaired object manipulation and grasping. At the time of recording, the patient was receiving clonazepam, with partial reduction in startle intensity, although responses persisted (Figure 1).

## 2 Discussion

Our case highlights hyperekplexia as an important differential diagnosis in neonates presenting with seizure‐like paroxysmal events. Although sustained generalized stiffness is traditionally considered a hallmark feature, increasing clinical and genetic characterization has demonstrated broader phenotypic variability, including presentations in which exaggerated startle responses predominate in the absence of persistent stiffness [2, 3, 4, 7]. In our patient, the presence of a markedly exaggerated, non‐habituating startle reflex with head retraction, despite the absence of sustained generalized stiffness, supports this expanding clinical spectrum of GLRB‐related hyperekplexia.

The initial neonatal presentation was clinically interpreted as a possible seizure, leading to acute treatment with phenobarbital despite the absence of an electroencephalographic ictal correlate. This scenario illustrates a well‐recognized diagnostic challenge in neonatal neurology, as exaggerated startle responses in hyperekplexia may closely resemble epileptic events [4, 5, 6]. Distinguishing epileptic from nonepileptic paroxysmal neonatal events may be particularly difficult during the neonatal period, when semiology is often limited and clinical manifestations may overlap [5, 6].

Although hyperekplexia is classically considered a nonepileptic disorder, epileptic seizures and epileptiform EEG abnormalities have been reported in a subset of patients with GLRB‐related disease [7, 8, 9]. In our patient, later electroclinically confirmed focal seizures were phenomenologically distinct from the exaggerated stimulus‐sensitive startle responses observed during the neonatal period. This overlap may further complicate the electroclinical interpretation of neonatal paroxysmal events and reinforces the importance of careful longitudinal assessment and EEG evaluation in patients with atypical or evolving clinical manifestations.

Recognition of characteristic features, including stimulus‐triggered exaggerated startle responses and head retraction, may help support the diagnosis of hyperekplexia and avoid unnecessary or suboptimal treatment. In this context, careful neurological examination and video‐based assessment remain particularly valuable diagnostic tools.

Whole genome sequencing identified a homozygous truncating GLRB variant (c.634C>T; p.Arg212Ter), previously reported in individuals with hyperekplexia [1, 7]. According to the *American College of Medical Genetics and Genomics and the Association for Molecular Pathology* criteria [10], the variant was classified as pathogenic. The variant introduces a premature stop codon and is predicted to result in loss of normal protein function, consistent with the established loss‐of‐function mechanism underlying GLRB‐related hyperekplexia [10, 11, 12]. The presence of the variant in the homozygous state, together with the characteristic exaggerated startle phenotype observed in our patient, strongly supported the molecular diagnosis.

From a therapeutic perspective, clonazepam remains the treatment of choice for hyperekplexia because of its enhancement of inhibitory neurotransmission [2, 3, 7]. In contrast, conventional antiseizure medications may have limited benefit for nonepileptic startle events, highlighting the clinical importance of early recognition and accurate diagnostic distinction. Although our patient later developed epileptic seizures adequately controlled with phenobarbital, the persistent exaggerated startle responses showed partial improvement only after the introduction of clonazepam.

Long‐term neurodevelopmental outcomes in GLRB‐related hyperekplexia appear variable, ranging from relatively preserved development to significant motor and cognitive impairment [1, 7, 8, 9]. Feeding difficulties, hypotonia, developmental delay and behavioural abnormalities have been described, particularly in individuals with severe neonatal presentations [1, 7, 8]. The marked motor delay and feeding impairment observed in our patient at 9 months of age are consistent with the more severe end of this clinical spectrum and highlight the importance of early recognition and multidisciplinary follow‐up.

These findings reinforce the importance of considering hyperekplexia in neonates presenting with seizure‐like paroxysmal events, particularly when electroencephalographic findings are inconclusive and exaggerated stimulus‐sensitive responses are observed. The absence of sustained generalized stiffness may further complicate recognition and contribute to diagnostic delay.

Early identification of hyperekplexia is clinically relevant because appropriate treatment may substantially reduce startle responses and improve patient management. Careful neurological examination, recognition of characteristic clinical features and complementary video assessment may facilitate earlier diagnosis and prompt targeted genetic investigation in atypical neonatal presentations.

## 3 Learning Points

- Hyperekplexia should be considered in neonates presenting with seizure‐like paroxysmal events, particularly when electroencephalographic findings are inconclusive.
- Careful neurological examination and video‐based assessment may facilitate early recognition and targeted genetic investigation in neonatal startle disorders.
- Persistent generalized stiffness may be absent in genetically confirmed GLRB‐related hyperekplexia.

## Funding

The authors have nothing to report.

## Ethics Statement

The authors declare that the research presented in this manuscript adheres to the ethical principles of the Ribeirão Preto Medical School, University of São Paulo, and the Declaration of Helsinki (1964), as revised in 2013. Written informed consent for publication was obtained from the patient's legal guardians.

## Consent

Written informed consent for publication of this case report, including clinical information, images, and video material, was obtained from the patient's legal guardians. Every effort was made to protect patient anonymity.

## Conflicts of Interest

The authors declare no conflicts of interest.

## Supporting information


**Video S1:** Nine‐month‐old patient demonstrating exaggerated, non‐habituating startle responses to tactile stimuli associated with head retraction and generalized hypotonia.

**Figure S1:** Representative frame from the supplementary video.

## Data Availability

The data that support the findings of this study are available from the corresponding author upon reasonable request.
